# The Big Data Science Center at the Shanghai Synchrotron Radiation Facility: the architecture of the superfacility

**DOI:** 10.1107/S1600577526005795

**Published:** 2026-06-22

**Authors:** Xiaoyun Li, Chunpeng Wang, Jige Chen, Rongzheng Wan, Jing Ye, Qintong Li, Haojun Cai, Ying Zhang, Aiguo Li, Renzhong Tai, Alessandro Sepe

**Affiliations:** a Shanghai Synchrotron Radiation Facility Shanghai Advanced Research Institute, Chinese Academy of Sciences No.239 Zhangheng Road Shanghai201210 People’s Republic of China; b Information Department Shanghai Advanced Research Institute, Chinese Academy of Sciences No. 239 Zhangheng Road Shanghai201210 People’s Republic of China; Bhabha Atomic Research Centre, India

**Keywords:** synchrotron, big data, artificial intelligence, centralized platform, superfacility platform, multidisciplinary, user-centric

## Abstract

In the context of the global big data deluge, concerted efforts are being made to address the challenges faced by large scientific facilities. These efforts are focused on providing users with the full potential offered by real-time, remote and self-driving experiments, where AI-driven analysis can guide experimental decisions in real time, while ensuring that the data pipelines adhere to the findability, accessibility, interoperability and reuse principles throughout their entire facility lifecycle. Besides all the efforts being made, a user-centric and user-friendly centralization of the overall scientific computing framework at the large scientific facilities remains a work in progress. To address this challenge the Big Data Science Center at the Shanghai Synchrotron Radiation Facility has developed and deployed a centralized, cohesive and user-friendly platform on top of its already existing superfacility framework, which is designed to manage the complete data lifecycle at large scientific facilities. This user-centric platform has transformed the user experience, shifting focus from complex data operations to scientific interpretation. Consequently, the accessibility of the facility to users has been considerably enhanced, thereby expediting the pace at which their discoveries are made.

## Abstract 2

The Big Data Science Center at the Shanghai Synchrotron Radiation Facility has developed a user-friendly centralized superfacility platform that integrates the entire large scientific facility lifecycle into a single accessible solution for users, while ensuring that the data lifecycle remains compliant with the findability, accessibility, interoperability and reuse principles. By leveraging AI-enabled big data frameworks, this unified user-centric platform transforms user experience by shifting focus from complex operations to scientific discovery, thereby considerably enhancing the accessibility of the facility to users and accelerating the pace of their discoveries.

## 1. Introduction

Current efforts in synchrotron facilities are converging along two major strategic directions. The first is experiment-centric, focusing on streamlining the entire experimental workflow from device control and detector data acquisition to real-time processing, visualization and storage, often achieved through highly optimized control systems combined with data-flow or data-broker. The second is data-centric, which prioritizes the full data lifecycle, from data acquisition and high-performance computing (HPC)-powered analysis to data archiving and sharing, often built upon integrated data management systems. While many leading facilities worldwide have historically emphasized the experiment-centric approach through advanced beamline control and online processing frameworks, initiatives such as the Big Data Science Center (BDSC) at Shanghai Synchrotron Radiation Facility (SSRF) exemplify the data-centric paradigm.

In recent years, with the development of complementary metal-oxide-semi-conductor and charge-coupled devices, the technology of detectors has developed rapidly. Nowadays, new hybrid photon-counting detectors with a very short readout time (nanoseconds) and a very small pixel size (down to 55 µm^2^) are widely used at different synchrotron beamlines, including the DECTRIS PILATUS and EIGER detectors, as well as the Tristan detector developed at Diamond Light Source (Förster *et al.*, 2019 ▸; Yang *et al.*, 2022 ▸; Weng *et al.*, 2023 ▸; Fardin *et al.*, 2023 ▸; Desjardins *et al.*, 2021 ▸; Taguchi *et al.*, 2008 ▸; Dectris, 2026 ▸; Omar *et al.*, 2022 ▸; Crevatin *et al.*, 2015 ▸). These detectors are, thus, capable of collecting data with a high data rate and high resolution; therefore, they are especially suitable for time-resolved and *in situ* experiments at the synchrotron facilities. However, the development of new synchrotron techniques, combined with the steady increase, in terms of brightness and flux, of the synchrotron beam, brings an unprecedented challenge to all the large facilities worldwide—the big data deluge—consisting of a steep increase (orders of magnitude) in the production of raw data (Wang *et al.*, 2018 ▸). This is a huge challenge for scientific computing at large facilities, affecting the entire facility lifecycle, including data storage, data transfer, data analysis and data management, and, in turn, the multidisciplinary scientific productivity of users. This encompasses a range of significant scientific disciplines, including chemistry, physics, energy and material science, medical and pharmaceutical science, bioscience, electronics, environmental sciences, archeology and manufacturing, while affecting the overall productivity of the entire facility (Wang *et al.*, 2018 ▸). Besides the data deluge challenge, at the large scientific facilities there are more challenges that the users need to overcome in order to successfully carry out their research. Traditionally, in fact, at the beamlines, the users are presented with a set of very basic manual protocols and data reduction and analysis software, which demands for users and facility staff to be fully engaged with an intense manual labor, which is prone to human mistakes, and does not guarantee optimal final results. Furthermore, traditionally, after the beam time, the users manage their collected data using public workstations on the premises, which raises concerns regarding data privacy and security. Moreover, once the users are back at their home institutions, they spend months analyzing their data using basic and manual data management and data analysis methods and software (Parkinson *et al.*, 2016 ▸). Scientific computing is thus proposing a new paradigm at the large scientific facilities worldwide, aiming at addressing these issues while targeting a dramatic increase in the users’ scientific productivity and facility efficiency. This includes the introduction at the large scientific facilities of massive parallelization, as well as novel computing architectures capable of enabling remote, real-time and high-throughput experiments (Blaiszik *et al.*, 2019 ▸; Wang *et al.*, 2001 ▸). Meanwhile, the traditional computing infrastructures at the large scientific facilities are not able to handle the big data deluge (Wang *et al.*, 2001 ▸); they are also not fit to scale-up experimental data workflows and pipelines at the modern synchrotrons, as well as not suitable to manage the big data synchronization required to efficiently handle the most different data sources at the last-generation synchrotron facilities (Parkinson *et al.*, 2016 ▸). Additionally, they lack a unified data management system, which effectively limits the amount of the experimental data that a user can investigate. Therefore, the development of a centralized user-friendly software platform, capable of efficiently managing and analyzing in real time the big data produced at the high-throughput synchrotron facilities, is an urgent and timely need. Once this is achieved, users can thus verify in real time the quality of the data they have collected, and, if necessary, adjust the experimental set-up directly during their beam time, thus dramatically optimizing their final scientific results. By leveraging state-of-the-art big data and artificial intelligence (AI), scientific computing approaches can further facilitate the operations of synchrotron users. This addresses the crucial need for a fully automated experimental pipeline and data management system, thereby significantly reducing the occurrence of human errors due to manual labor. Consequently, users can focus exclusively on their scientific research, leading to an increase in the number of new scientific discoveries.

Recently, efforts have been made worldwide in order to address the big data deluge challenges at the large scientific facilities (Basham *et al.*, 2015 ▸; Atwood *et al.*, 2015 ▸; Diamond Light Source, 2017 ▸; Filik *et al.*, 2017 ▸; Donatelli *et al.*, 2015 ▸; Pandolfi *et al.*, 2018 ▸; Parkinson *et al.*, 2017 ▸; Chard *et al.*, 2019 ▸; Li *et al.*, 2021 ▸; Liu *et al.*, 2022 ▸; Diamond Light Source, 2022 ▸; Pedersen *et al.*, 2013 ▸). In fact, major synchrotron facilities worldwide have been developing sophisticated computational and AI infrastructures to address the big data deluge challenges. Furthermore, the Globus cloud-hosted data automation services for large scientific facilities have been developed in order to provide a robust, automatized and remote computing infrastructure for data management and data analysis (Blaiszik *et al.*, 2019 ▸; Chard *et al.*, 2023 ▸; Deslippe *et al.*, 2014 ▸; Gürsoy *et al.*, 2014 ▸; Park *et al.*, 2015 ▸). This solution offers a standardized, secure, highly efficient approach to remotely accessing and processing scientific datasets (Chard *et al.*, 2014 ▸). The Advanced Photon Source (APS) has established a comprehensive data management ecosystem through the APS Data Management System (Veseli *et al.*, 2018 ▸), which integrates with the Argonne Leadership Computing Facility (ALCF) to enable automated processing workflows. This architecture leverages Globus services to orchestrate data movement and computation across geographically distributed resources (Parraga *et al.*, 2023 ▸). Similarly, the European Synchrotron Radiation Facility (ESRF) has deployed DRAC, a scalable data repository built upon the ICAT metadata catalog framework that manages both raw and processed data while adhering to the Findability, Accessibility, Interoperability and Reuse (FAIR) principles (De Maria *et al.*, 2024 ▸). The ESRF–EBS upgrade necessitated a complete revision of the data acquisition tools, thus resulting in the BLISS experiment control system, the LIMA2 for high-speed detector control, and the EWOKS workflow system for automated data reduction (Meyer *et al.*, 2023 ▸). The National Synchrotron Light Source II (NSLS-II) has developed an integrated infrastructure centered on the BlueSky data acquisition framework, which has been adopted across multiple facilities including APS and LCLS (Pouchard *et al.*, 2019 ▸). Recent infrastructure upgrades have enabled seamless remote access through the JupyterHub and Guacamole framework, facilitating mail-in experiments without requiring VPN connectivity (Rakitin *et al.*, 2022 ▸). Diamond Light Source, alongside the Science and Technology Facilities Council (STFC), has contributed significantly to the ICAT collaboration, developing standardized metadata catalogs that support cross-facility data discovery and retrieval (Götz *et al.*, 2023 ▸). The Advanced Light Source (ALS) employs the SPOT suite for data management, transferring datasets to the National Energy Research Scientific Computing Center (NERSC) for archival and processing (Antypas *et al.*, 2021 ▸). A critical advancement across these facilities is the implementation of automated reconstruction pipelines powered by machine learning. The APS has demonstrated AI-enabled Bragg diffraction analysis using BraggNN, achieving speed improvements of over 200 times compared with conventional methods, while the TomoGAN framework enables low-dose tomographic reconstruction (Parraga *et al.*, 2023 ▸). ESRF’s EWOKS system supports online tomography reconstruction and EXAFS visualization during acquisition, with recent implementations enabling autonomous experiments through machine-learning-based feedback loops (Meyer *et al.*, 2023 ▸). Cross-facility workflows have been realized through the Globus platform and Gladier toolkit, allowing experimental data collected at facilities like APS to be processed remotely at ALCF using specialized AI accelerators such as Cerebras with turnaround times under 30 s (Liu *et al.*, 2021 ▸; Vescovi *et al.*, 2022 ▸).

Remote access capabilities have evolved substantially, with facilities deploying web-based portals that provide secure data browsing, reprocessing and visualization without requiring local computing resources. The integration of identity management systems, multi-factor authentication and ScienceDMZ network architectures ensures both security and high-performance data transfer (Rakitin *et al.*, 2022 ▸; Parraga *et al.*, 2023 ▸). These developments collectively establish a foundation for self-driving laboratories where AI-driven analysis can guide experimental decisions in real time, maximizing the scientific output of limited beam time while ensuring that the data pipelines adhere to the FAIR principles throughout their entire lifecycle.

Besides all the efforts being made, the centralization of the overall scientific computing framework at the large scientific facilities, aimed at delivering a facility-wide, friendly, immersive, efficient and productive user experience, remains a work in progress. The SSRF has been equally affected by the big data deluge and by the lack of a centralized scientific computing infrastructure, where each beamline was using its own local and limited computing resources to store and crudely process their users’ experimental datasets.

To address these challenges, the SSRF has established, as a part of the SSRF Phase II Upgrade project, the BDSC, which has planned, designed, developed, deployed, and now operates and upgrades, a next-generation scientific computing architecture at the SSRF, thus creating the first superfacility in China, and one of the first worldwide (Wang *et al.*, 2021 ▸). At SSRF, our strategy centers on implementing a structured and systematic approach to address the multifaceted challenges of the big data deluge. This is realized through a cohesive architecture designed to manage the complete data lifecycle. A high-performance network backbone coupled with a unified, scalable storage system forms the foundation, ensuring efficient transmission and reliable long-term preservation of high-throughput data pipelines. To meet the demand for rapid online processing and analysis, a hybrid HPC infrastructure, integrating both CPU and GPU clusters, provides the necessary computational power. Moreover, for secure archival and backup, a hierarchical storage architecture incorporating a robotic library system is employed, guaranteeing data integrity and accessibility. Furthermore, SSRF has been upgraded with 38 beamlines and 52 experimental endstations, with nearly 100 users per day accessing the SSRF, and producing approximatively 45 PB of raw data per year (Tai & Zhao, 2024 ▸). Following the SSRF upgrade, SSRF is now able to support one of the largest plethora of the most diverse disciplines worldwide. Thus, the BDSC platform will be pivotal in opening the synchrotron facility to non-expert users, with limited, if any, experience in terms of synchrotron studies. Besides, real-time experiments are going to be increasingly central at the SSRF. Therefore, addressing their big data challenges through the implementation of novel HPC architectures tailored to the SSRF users’ scientific needs (*e.g.in situ* real-time structural analysis, imaging, 3D reconstructions, structural biology, macromolecular investigations, material science simulations and modeling, structural molecular-based catalysts, electrocatalyst structure-activity relationship, finite element analysis, *etc*.), is of a crucial importance (Parkinson *et al.*, 2017 ▸; Tao *et al.*, 2021 ▸; Lutter *et al.*, 2021 ▸; Ebrahimi-Kahrizsangi *et al.*, 2015 ▸; Wang *et al.*, 2021 ▸; Ye *et al.*, 2023 ▸). Despite these significant advances, the BDSC big data framework did not incorporate a user-centric superfacility platform to consolidate the entire large-scale facility lifecycle into a single, accessible, user-friendly solution for all the users. Such a platform would deliver a unified access for users, thereby integrating scientific computing, big data architectures, HPC, and data science capabilities across their entire research workflow, from proposal submission to final publication, while ensuring FAIR-compliant data stewardship and AI empowerment. Therefore, since 2021, SSRF has significantly augmented its core infrastructure. Storage capacity has been expanded approximately fourfold, while aggregate computing power has increased roughly fivefold, providing a substantially more robust foundation for data-intensive operations. This period of enhancement has also seen a comprehensive extension of capabilities across the entire data lifecycle. Recognizing the asymmetrical and highly concurrent nature of experimental data workflows, we have architected and optimized an edge-cloud collaborative framework. This distributes processing tasks, alleviating central network and computational loads while enabling low-latency preliminary analysis at the beamline. Crucially, a unified identity authentication, authorization and access control system is rigorously enforced across the entire SSRF lifecycle, ensuring robust data security and user privacy. These technical components are now seamlessly integrated into a single unified user platform, the Artificial Intelligence SSRF Superfacility Platform (AI-SSRF-SP), centralizing data management, integrating heterogeneous computational resources, and enforcing the FAIR principles across the entire AI-enabled facility, thereby bridging the superfacility’s experimental operations with its large-scale data infrastructure, yielding a unified solution targeted at enhancing users’ scientific productivity and collaboration. This unified user portal consolidates data management, computational resource scheduling, and auxiliary laboratory services, while enabling remote experiment capabilities. Collectively, this integrated ecosystem transforms the user experience, shifting focus from complex operations to scientific interpretation and significantly accelerating the pace of discovery. Furthermore, the AI-SSRF-SP has been designed to address complex multimodal experiments, as well as to facilitate the establishment of a shared scientific metadata and raw data repository.

Thus, this article details the architecture, pipelines, software, hardware, infrastructure, remote access and automation constituting the AI-SSRF-SP. It details the planning, design, development and deployment of the AI-SSRF-SP by the BDSC, exploring its meticulous design and specific tailoring to enhance the user’s scientific experience and productivity. This article also demonstrates how the AI-SSRF-SP has significantly increased user accessibility to AI-enabled large-scale scientific computing frameworks and infrastructures, which would otherwise remain highly under-utilized. It provides an overview on the upgraded scientific computing resources and infrastructure available to the SSRF users, as well as on the AI-SSRF-SP, which has enabled the centralization and unification of the entire SSRF data lifecycle within a single solution seamlessly accessible by the users. Furthermore, it describes the BDSC endeavors to automate the scientific data pipelines facility-wide with the objective of enhancing user productivity, integrating the innovative users’ science carried out at the SSRF beamlines with the BDSC advanced scientific computing architecture. This effectively supports the SSRF users navigating throughout the big data deluge at the large scientific facilities, thereby significantly increasing their scientific output.

## 2. Hybrid edge-cloud HPC architecture

**Figure 1:**
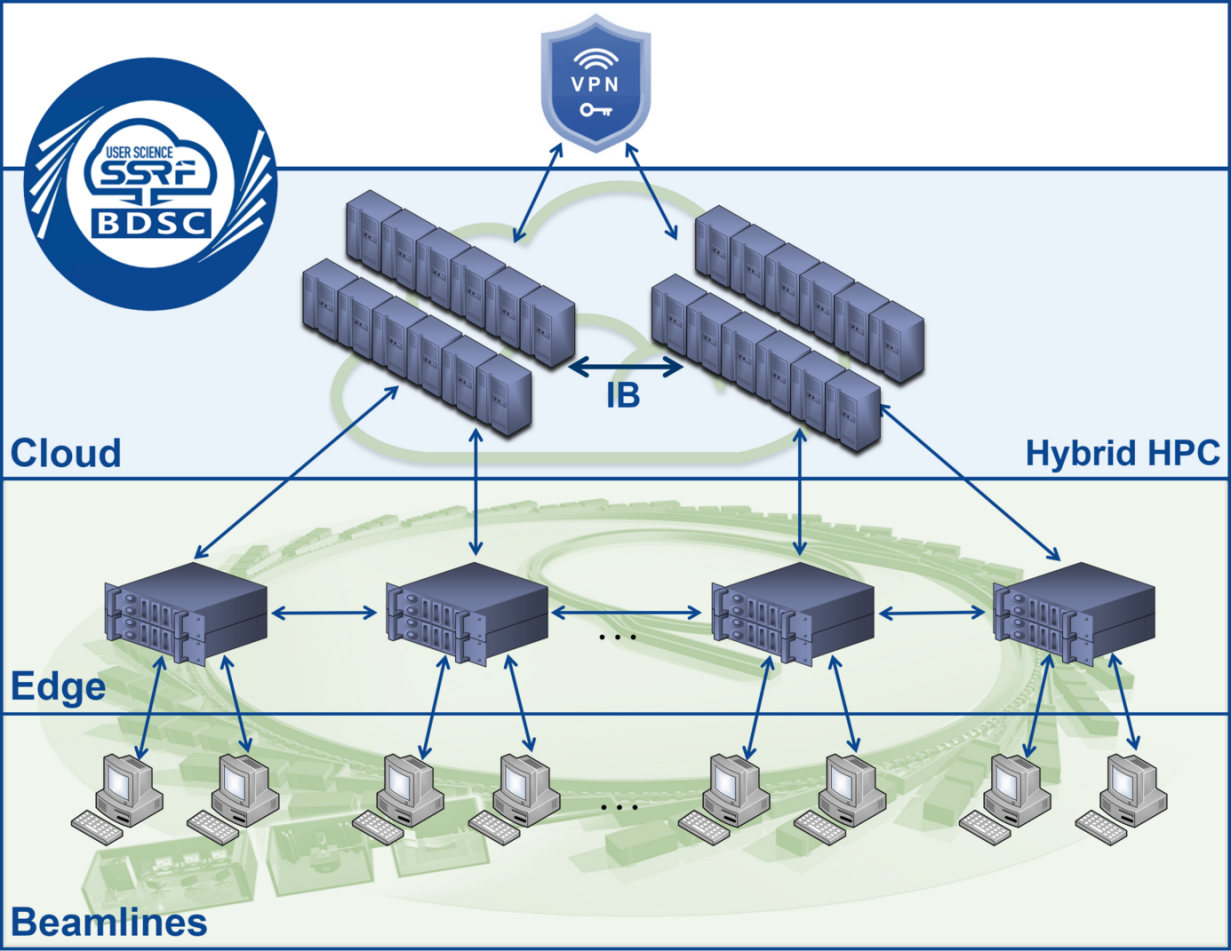
The BDSC scientific computing architecture. The BDSC HPC network backbone is powered by InfiniBand (IB).

The BDSC aims at supporting all the SSRF beamlines and users via a state-of-the-art scientific computing infrastructure (Fig. 1 ▸), from thin terminals equipped with user-friendly graphical user interfaces (GUIs) to big data platforms to remote experiments to virtualization to AI to automation to hybrid edge/cloud HPC to robust high speed storage systems and low latency networks. The BDSC provides the SSRF users and beamlines with massive parallelization, supporting unified data pipelines, and resulting in a centralized data storage, management, processing, analysis, interpretation and subsequent result visualization. Additionally, the BDSC has developed and deployed automated data pipelines facility-wide at the SSRF, which enabled the users at the SSRF to access real-time data evaluation during their beam times. The following sub-sections herein detail the novel hybrid edge-cloud HPC backbone that has been deployed by the BDSC to support its AI-SSRF-SP.

### 2.1. High-throughput data pipelines and massive storage systems

Prior to the implementation of a unified data management system across all beamlines at the SSRF, each individual beamline was equipped with its own local data storage system. However, the absence of a comprehensive management system capable of delivering authentication functionalities, while enforcing policies and access control for user data, resulted in a significant security risk. To address this crucial issue, and the growing pressure on the former data storage system, unable to accommodate the new scientific computing needs due to the entire SSRF facility now being fully connected to the BDSC, a unified Network Attached Storage (NAS) parallel distributed storage system was planned and deployed by the BDSC. This connects all the beamlines at the SSRF, while storing all the users’ experimental raw data, metadata and processed data. Furthermore, to address the critical challenge of establishing a unified, secure and high-performance data backbone, capable of managing the high-throughput data flow from diverse beamline detectors, each experimental station has been equipped with an access switch, which is connected to two 40 Gb s^−1^ core switches. This resulted in experimental pipelines capable of transferring data from the detectors at the beamlines to the BDSC with a data rate of 70 MB/s (see Section S1 of the supporting information).

The implementation of this parallel distributed storage architecture, featuring a unified namespace and robust redundancy, represents a significant infrastructural advancement, achieving reliable, high-availability storage at multi-petabyte scale and seamless cross-platform access for users.

### 2.2. Robotic data archiving and backup

To addresses the challenge of ensuring the long-term preservation, integrity, and recoverability of the massive experimental datasets generated at SSRF, which extends beyond the capabilities of online storage systems, the BDSC has implemented the deployment of an enterprise-class automated robotic tape archiving system that enables scalable, policy-based archival with high reliability and intelligent management, establishing a robust foundation for the large facility’s centralized data backup and disaster recovery strategy (see Section S2).

### 2.3. Online real-time user data analysis

To provide a solution to the computational bottleneck imposed by the need for online real-time analysis of massive experimental datasets during user beam time, the BDSC has implemented an upgrade to its previous HPC infrastructure. This comprises a novel and heterogeneous HPC computing infrastructure, beyond traditional resources, equipped with hybrid CPU/GPU clusters, fat nodes, cross-platform servers and graphics workstations. Thus, the overall BDSC HPC infrastructure is now capable of providing SSRF users with access to 208 CPU nodes, 13 GPU nodes, 3 FAT nodes, 11512 CPU cores and 60 GPU graphics cards, capable of nearly 2.2 PFlop s^−1^. The BDSC HPC clusters are configured with four servers as login nodes. The login nodes are the entrance for the users to access the clusters’ computing functionalities. They allow the users to log in to the system and apply for resources, as well as for submitting and querying their computing jobs. The clusters are configured with two servers as management nodes. Management nodes are used to run the cluster management software and perform critical tasks, including job scheduling and cluster monitoring (see Section S3). The BDSC clusters are based on the Linux Red Hat 64-bit operating system. Table 1 ▸ summarizes the software environment that the BDSC provides to its users.

The implementation of this large-scale hybrid CPU/GPU HPC cluster upgrade, delivering petaflop performance with high operational efficiency, represents a substantial leap in on-site computational capability, directly empowering users with the accelerated processing required for demanding scientific workflows.

### 2.4. Facility-wide asymmetric and high-concurrency computing architecture

**Figure 2:**
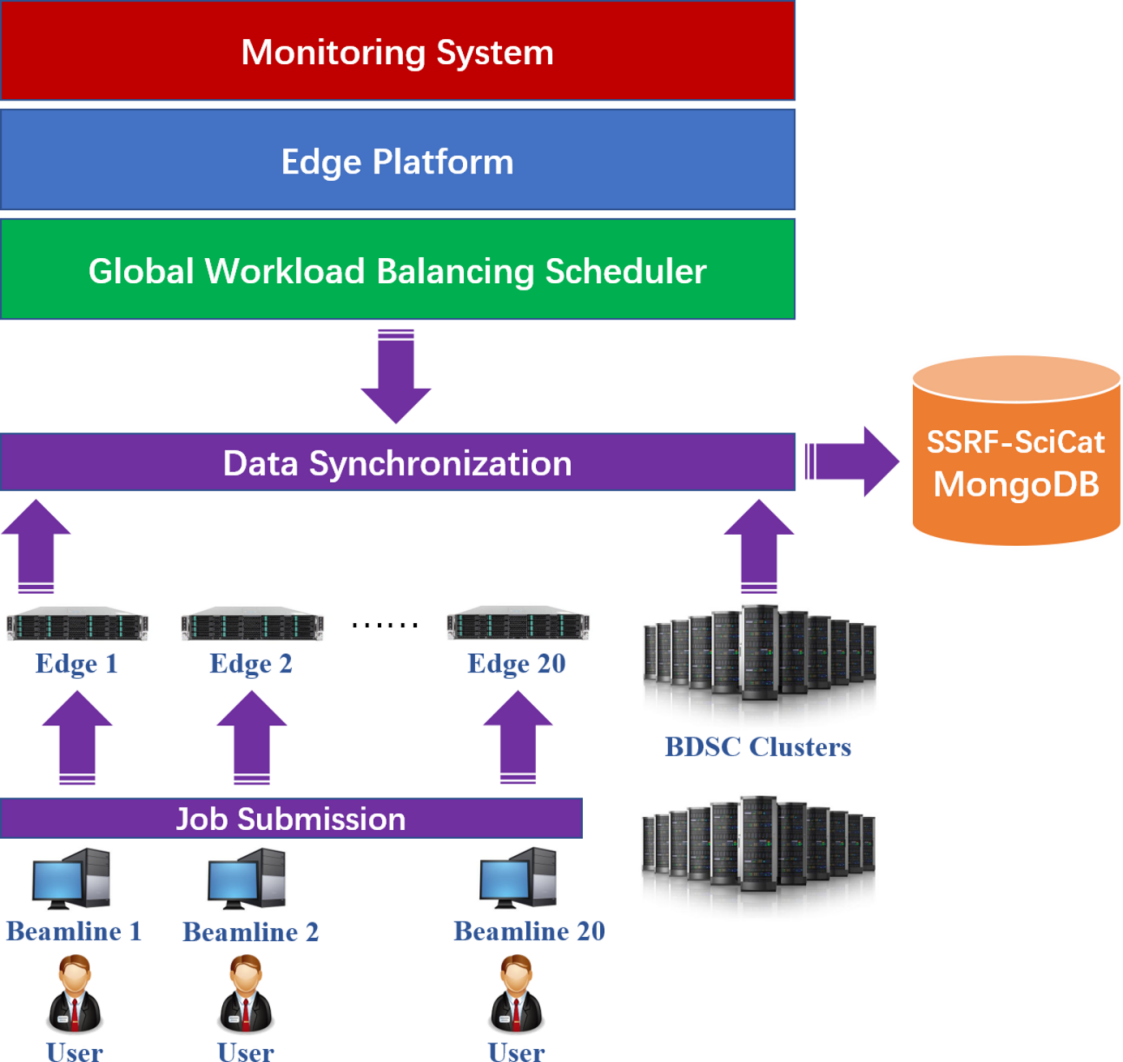
The BDSC edge infrastructure at the SSRF.

In addition to the previous scientific computing approach (Wang *et al.*, 2021 ▸), in which the beamlines were connected through direct pipelines or edge cluster pipelines to the BDSC, the BDSC has now introduced a substantial enhancement to address the complex challenge posed by the highly asymmetric and concurrent computational workloads across the most diverse beamlines. This is a scenario with which traditional centralized architectures struggle to manage efficiently without introducing network bottlenecks or single points of failure. The BDSC has implemented a sophisticated architecture where experimental data at the SSRF are automatically and remotely transferred, through dedicated HPC pipelines, from the edge clusters, deployed by the BDSC at the beamlines, to the BDSC clusters, via a high-speed network (Fig. 2 ▸). This architecture has been devised in order to optimize the workload balancing, while increasing the efficiency and reliability, of the data transferred and processed at the SSRF from the beamlines to the BDSC, eliminating any risk connected to a single point of failure, which may seriously hinder the users’ experiments and the beamlines operations. A total of 20 edge clusters have been deployed by the BDSC, ensuring comprehensive coverage of all the SSRF beamlines, with a storage capacity of ∼1 PB, accounting for two weeks of data storage, and augmented with dedicated data processing capabilities, tailored, at the edge, for each beamline (see Section S4). The edge clusters provide the beamlines with a local buffer and an emergency backup mechanism, while reducing the intensive workload pressure on the network and on the BDSC cloud. To date, 20 beamlines utilize the hybrid HPC framework directly, whereas 12 employ the edge pipelines, and 6 beamlines have been configured to connect directly to the BDSC cloud via dedicated pipelines. The diverse solutions are not contingent upon architectural constraints, rather primarily dictated by the volume of experimental data effectively produced by each beamline, with the hybrid HPC framework being employed in cases of high volume and the edge pipelines being sufficient in cases of lower volume. Furthermore, the BDSC has planned, designed, developed and deployed, as well as, now, operating and upgrading, a platform capable of managing the storage and the computing pipelines connecting the edge clusters at the beamlines with the entire BDSC scientific computing infrastructure at the SSRF. This platform is capable of workload balancing and data synchronization among the edge clusters, and between them and the BDSC clusters, while providing unified resource scheduling services for the edge and BDSC computing nodes, as well as feeding the metadata into the SSRF-SciCat system (Wang *et al.*, 2021 ▸), and monitoring the status of edge clusters.

The BDSC’s accomplishment in the successful design and deployment of a coordinated cloud-edge hybrid computing framework represents a sophisticated architectural solution, effectively distributing processing loads, mitigating central infrastructure pressure, and ensuring system resilience while enabling low-latency preliminary analysis at SSRF.

### 2.5. User data privacy and security

To address the critical security and management challenges arising from fragmented identity sources and disparate access control systems, which in a multi-user, multi-institutional facility environment pose significant risks to data privacy and controlled resource utilization, the BDSC has developed and deployed the Unified User Interface & Management System (U^2^IMS) at the SSRF. The U^2^IMS employs an advanced unified authentication, permission management and access control system based on the OpenID Connect (OIDC) and the Open Authorization (OAuth) standards. This system is capable of transparently orchestrating users’ credentials from multiple authentication sources, both external and internal to the SSRF, including the Chinese Academy of Science (CAS) Large Research Infrastructures User Service Platform (LSSF), the SSRF and Shanghai Advanced Research Institute (SARI), through a convenient single sign-on approach.

The BDSC implementation of a unified authentication and authorization system represents a foundational security achievement, enabling secure, seamless single sign-on across heterogeneous internal and external identity providers while ensuring robust, policy-enforced data access control.

### 2.6. User-friendly integrated data service

**Figure 3:**
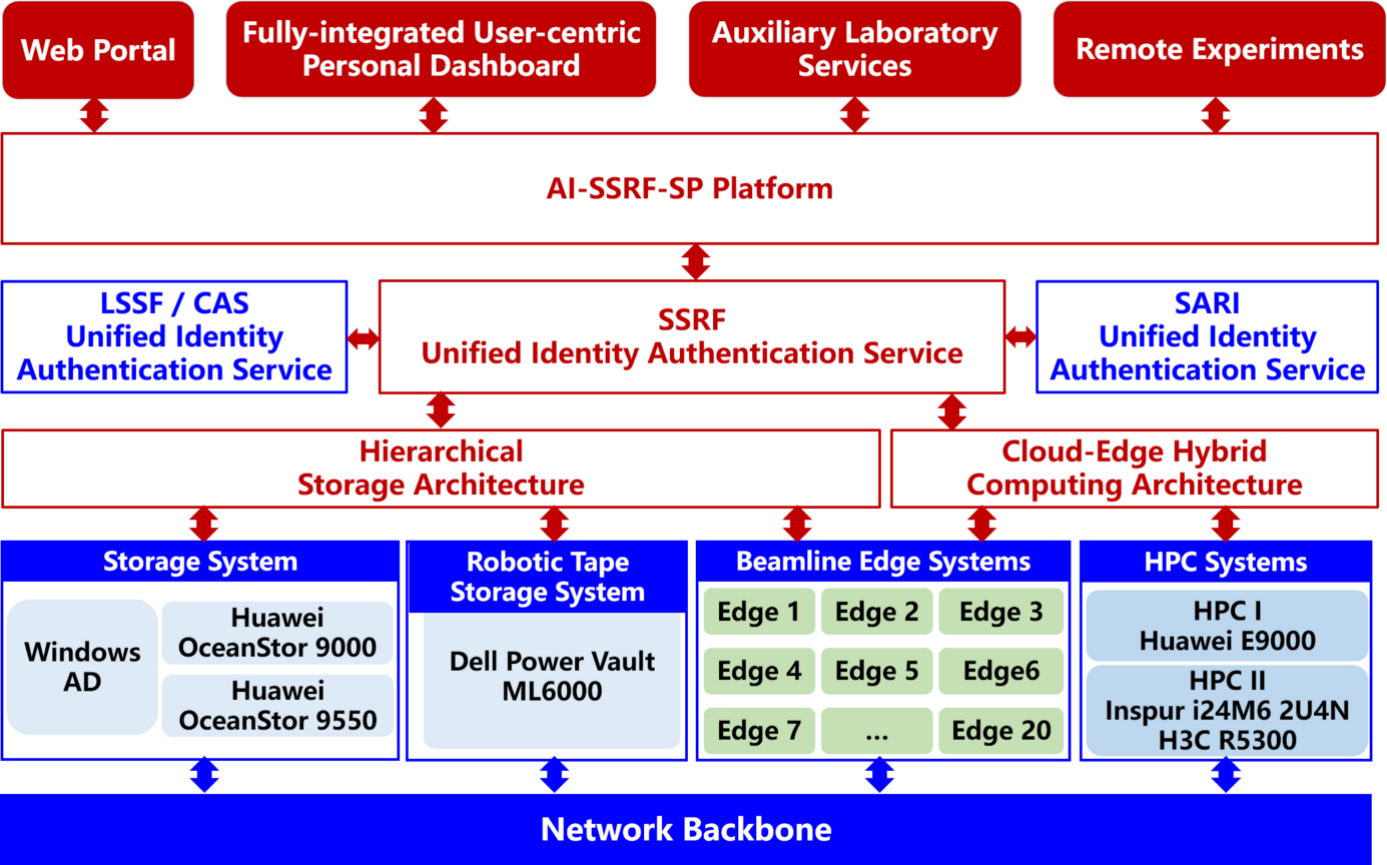
The AI-SSRF-SP architecture and workflow, as deployed by the BDSC at the SSRF.

To address the overarching challenge of integrating the most diverse and complex technical and scientific subsystems, ranging from data acquisition to computing and analysis, into a cohesive, user-centric service platform, thereby bridging the gap between advanced infrastructure and end-user scientific productivity, the BDSC has designed, developed and deployed the AI-SSRF-SP. The AI-SSRF-SP is a next-generation user-centric scientific computing platform targeting, facility-wide and nation-wide, data centralization, and encompassing the entirety of the SSRF beamlines (Fig. 3 ▸). It is accelerated by the BDSC hybrid edge/cloud HPC infrastructure and seamlessly integrated into the SSRF-SciCat. It aims at increasing the users’ scientific productivity, and its ensuing impact on technology advancements, unifying, standardizing, centralizing and automating the large facilities lifecycle, including users’ authentication systems, facility administration, monitoring and management systems and maintenance, as well as the entire users’ data lifecycle at the large scientific facilities, including data management and processing, software and hardware management, remote access and visualization, virtualization, proposals and publications management, supporting laboratories management, security audits, cluster resources scheduling, management and orchestration, beamline data processing pipelines, and several other services and facilities that the SSRF offers to its users.

The development and deployment of the AI-SSRF-SP platform represents the culmination of the superfacility architecture, successfully creating a unified, web-accessible portal that orchestrates the entire data and research lifecycle, significantly enhancing user experience, operational efficiency, and adherence to FAIR principles across the entire large facility.

### 2.7. Accessible experiments

To address the critical challenge of enabling geographically dispersed users to conduct and control experiments in real time, overcoming the traditional limitations of on-site presence which constrains accessibility and efficient utilization of specialized beamline facilities with limited beam time, the BDSC has deployed and integrated a virtual private network (VPN) system within the SSRF superfacility infrastructure. This allows SSRF users to carry out remote experiments at several of the SSRF beamlines, while allowing the SSRF engineering department to remotely access their relevant tasks and operations at the SSRF. To date, the beamlines integrated within the BDSC VPN framework, providing remote experiment capabilities, are BL02U1, BL10U2, BL13HB, BL09U, BL18U1 and BL19U1. Meanwhile, the beamlines that are integrated within the BDSC VPN framework, providing remote operations, are BL19U1, BL11B and the engineering department. The remote file transfer efficiencies of the BDSC VPN system are listed in Table 2 ▸.

The establishment of a secure, VPN-based remote access framework, supporting multiple beamlines with efficient data transfer capabilities, represents a pivotal enhancement in user service, and extending experimental opportunities beyond physical boundaries. In addition to the convenience of access to users during standard beam times, this may also result in a very compelling tool during times of international, national or facility lockdowns. This would guarantee that experimental operations are not interrupted by any cause of force majeure.

## 3. Implementation of the centralized superfacility platform

### 3.1. Beamline integration and online automatic data processing

To address the operational challenges of translating a centralized computational infrastructure into tangible, beamline-specific solutions, moving from generalized resource provisioning to the implementation of dedicated, automated data processing pipelines that deliver real-time analytical capabilities directly to users during experiments, the BDSC has successfully developed and deployed HPC pipelines connecting all the SSRF beamlines to its centralized architecture. Prime examples of fully automated data analysis pipelines established by the BDSC are the Biological Macromolecular Crystallography (MX) beamline (BL02U1), the Biosafety P2 Protein Crystallography (P2) beamline (BL10U2), the X-ray Imaging beamline (BL13HB), the Fast X-ray Imaging beamline (BL16U2) and the X-ray Nanotomography beamline (BL18B1). This enables users to automatically submit tasks, onsite, to the BDSC during their beam time, and the resulting data from their experiments at the SSRF can then be studied in real time. Ultimately, all the SSRF users’ raw data, metadata and final results are ingested by BDSC AI-SSRF-SP and integrated within the entire large facility data life cycle, including the users’ proposals and publications. In fact, following the BDSC design, development and deployment of tailored and dedicated automated HPC data pipelines, the beamlines at the SSRF experienced an increase in scientific productivity ranging up to a factor of 60 (Wang *et al.*, 2024 ▸). Furthermore, traditionally, experiments involving imaging and crystallographic methods require for the data to be processed, reconstructed and transferred back to the beamlines (Liu *et al.*, 2022 ▸; Gürsoy *et al.*, 2014 ▸; Yu *et al.*, 2019 ▸; Marone *et al.*, 2017 ▸). Consequently, the BDSC has architected, developed and deployed a dedicated and tailored data pipeline, capable of automatically interfacing the imaging and crystallography beamlines with the AI-SSRF-SP, while leveraging the automated cross-platform scientific HPC capabilities previously deployed by the BDSC (Wang *et al.*, 2021 ▸; Ye *et al.*, 2023 ▸; Wang *et al.*, 2024 ▸). Indeed, a significant accomplishment of the full implementation of the AI-SSRF-SP across the entire SSRF was the ability to maintain the scientific productivity acceleration previously attained by the BDSC via the deployment of its big data and metadata framework. It is possible for users to log in to the AI-SSRF-SP in real time and to access the experiment interface directly. By selecting the relevant client button for the experiment, the AI-SSRF-SP automatically transfers the project ID, experiment ID, passport account, and experiment username to the client. Following successful authentication, the client mounts the storage directory via the Server Message Block protocol in the edge disk directory, thereby checking and creating an experiment directory. The user then saves the dataset to a sub-directory of the experiment directory, and the client-side data collection program automatically synchronizes data to the BDSC. The AI-SSRF-SP is responsible for the analysis of all the datasets, the subsequent generation of the corresponding metadata list, and the subsequent feeding of this to SciCat. It supports HDF5, text, CSV and other relevant formats. The AI-SSRF-SP then enables users to access the metadata and raw data list for each of their experimental proposals.

Therefore, the BDSC was able to introduce remarkable and crucial features with its AI-SSRF-SP, which were previously absent at the SSRF. It also ensured the utmost security and confidentiality on all the users’ experimental data, authorizing the users to access, assess and analyze only their own data, while increasing the scientific productivity of the SSRF beamlines. In addition, the BDSC has integrated its previously developed and deployed SSRF-SciCat (Wang *et al.*, 2021 ▸; SciCat Project, 2026 ▸) within the broader AI-SSRF-SP, thereby enabling now all its integrated beamlines to benefit from its advanced metadata framework. This framework facilitates the integration of the entire SSRF metadata lifecycle with big data, AI and internet of things technologies, as well as with the beamline control system, data acquisition system, data analysis pipelines, storage, edge and HPC systems, facility-wide and nation-wide authentication systems, user proposal system and auxiliary laboratory management systems, among others. The endeavor was not without its challenges during the research and development phase, which was further compounded by the extensive use of disparate operating systems for experiments across the entire facility. For instance, compatibility issues were encountered when the client is opened on different browsers. Furthermore, the original client at the beamline was unable to distinguish between local and remote users, which resulted in issues with regard to security and traceability. Furthermore, the permission framework provided by the Microsoft Windows client was inadequate. In addition, more trivial issues were encountered as a result of the inherent design of the Microsoft Windows architecture, wherein drive letters experienced incompatibility with local resources. It was determined that the original disk array authentication system in place did not meet the rigorous security requirements of the AI-SSRF-SP. Moreover, it was necessary to resolve compatibility issues relating to the clients operating on the Linux operating systems. In order to address these issues, the original client was refactored into a background service process, which automatically runs after user login. Consequently, the browsers communicate with the client interface through a local URL. The client is now responsible for verifying the match between the location in which the experiment is carried out and the location in which the client is actually deployed. In the event of mismatching, access will be denied. The authentication system has been modified to enable the classification of users based on their authentication credentials, thereby restricting each user to access solely those AI-SSRF-SP functionalities pertaining to their categories. A framework was developed and deployed to automatically create and manage drive letter settings for each different beamline, thereby bypassing the operating system’s native limitations. The registry framework was further upgraded to accommodate multiple accounts for mounting edge storage. Furthermore, we have developed and deployed customized frameworks with the objective of expanding the capabilities of the operating systems at the local beamlines. Therefore, users are now able to access a unified and centralized platform that automatically fulfills all the specific requirements of each individual beamline, and transparently configures and makes available the BDSC resources to them, all within the same user-friendly interface. This has the effect of reducing the time taken to become proficient with the entire facility lifecycle, allowing users to focus more on their experiments and thus increasing their scientific productivity. It is now ensured that users can access their data, both locally and remotely, in accordance with the highest standards of security and confidentiality. This enhances the international competitiveness of the SSRF. Furthermore, users are now capable of accessing their data remotely and seamlessly via the AI-SSRF-SP, benefitting from a unified and centralized login system that offers an experience akin to that of users on site.

The deployment of tailored, automated HPC pipelines across multiple beamlines, resulting in order-of-magnitude accelerations in data processing and demonstrable multi-fold increases in scientific productivity, validates the superfacility architecture’s core premise of seamlessly integrating advanced computing with experimental workflows to drive user success.

### 3.2. The BDSC scientific cloud

To address the challenge of translating raw computational power into an accessible, versatile and scientifically productive cloud service that can cater to the heterogeneous demands of a multidisciplinary user community, moving beyond infrastructure provisioning to the delivery of integrated, application-ready research environments, the BDSC has further expanded the AI-SSRF-SP capabilities by building a scientific cloud directly inside its framework. This also includes the adoption of state-of-the-art virtualization and containerization technologies, which can now leverage the BDSC architecture that it is capable of handling data processing, reconstruction, modeling and analysis required by the 3D imaging methods, X-ray Detector Software (XDS) for crystal diffraction, molecular dynamics simulations, first principles calculation simulations and several other data-intensive scientific applications. Moreover, besides commercial and open-source software (Table 1 ▸), the BDSC also optimizes, augments, accelerates and integrates into its AI-SSRF-SP HPC scientific cloud several customized software, self-developed by the SSRF users, whom are granted access to the AI-SSRF-SP HPC scientific cloud based on their scientific requirements resulting from their SSRF research project.

**Figure 4:**
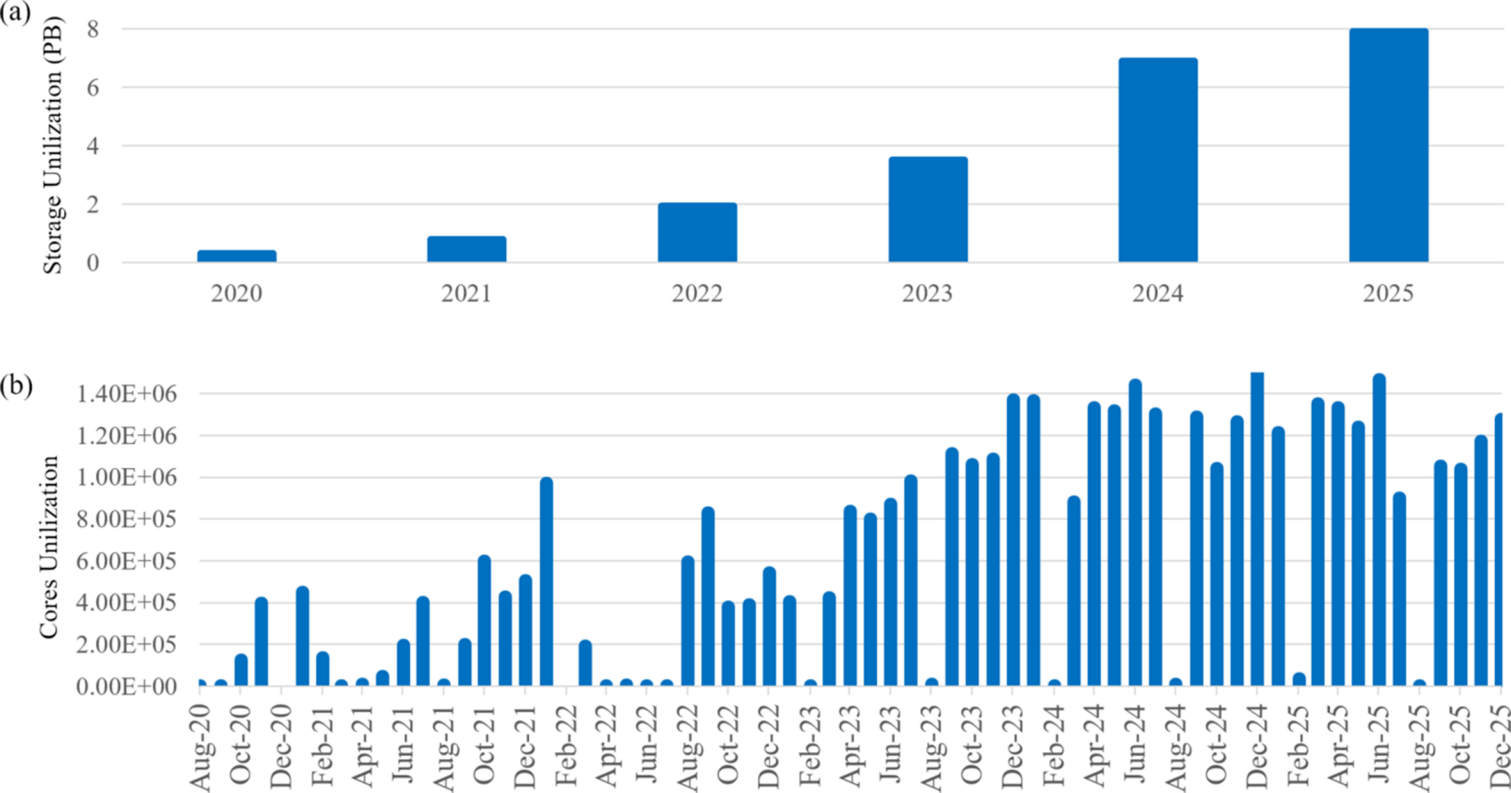
The BDSC annual (*a*) storage and (*b*) computing resources utilization.

To date, the BDSC has processed more than 1100000 scientific computing jobs. After commencing operations, the BDSC has been subject to a considerable degree of workload pressure from SSRF users, particularly in the fields of chemistry, physics, energy and material science, medical and pharmaceutical science, bioscience, electronics, environmental sciences, archeology and manufacturing. Indeed, the annual utilization of the BDSC storage and computing resources increased, on average, by ∼136% and 40%, respectively (Fig. 4 ▸). The sustained and significant annual growth in the users’ utilization of the resources provided by the AI-SSRF-SP, coupled with the successful support for diverse scientific jobs, robustly validates the efficacy and user-centric design and vision of the BDSC scientific cloud. This is a foundational pillar of the superfacility ecosystem with the objective of increasing the efficiency of the SSRF multimodal facility and the users’ multidisciplinary scientific productivity through the adoption of state-of-the-art big data, AI and robotic automation technologies.

### 3.3. AI-SSRF-SP

**Figure 5:**
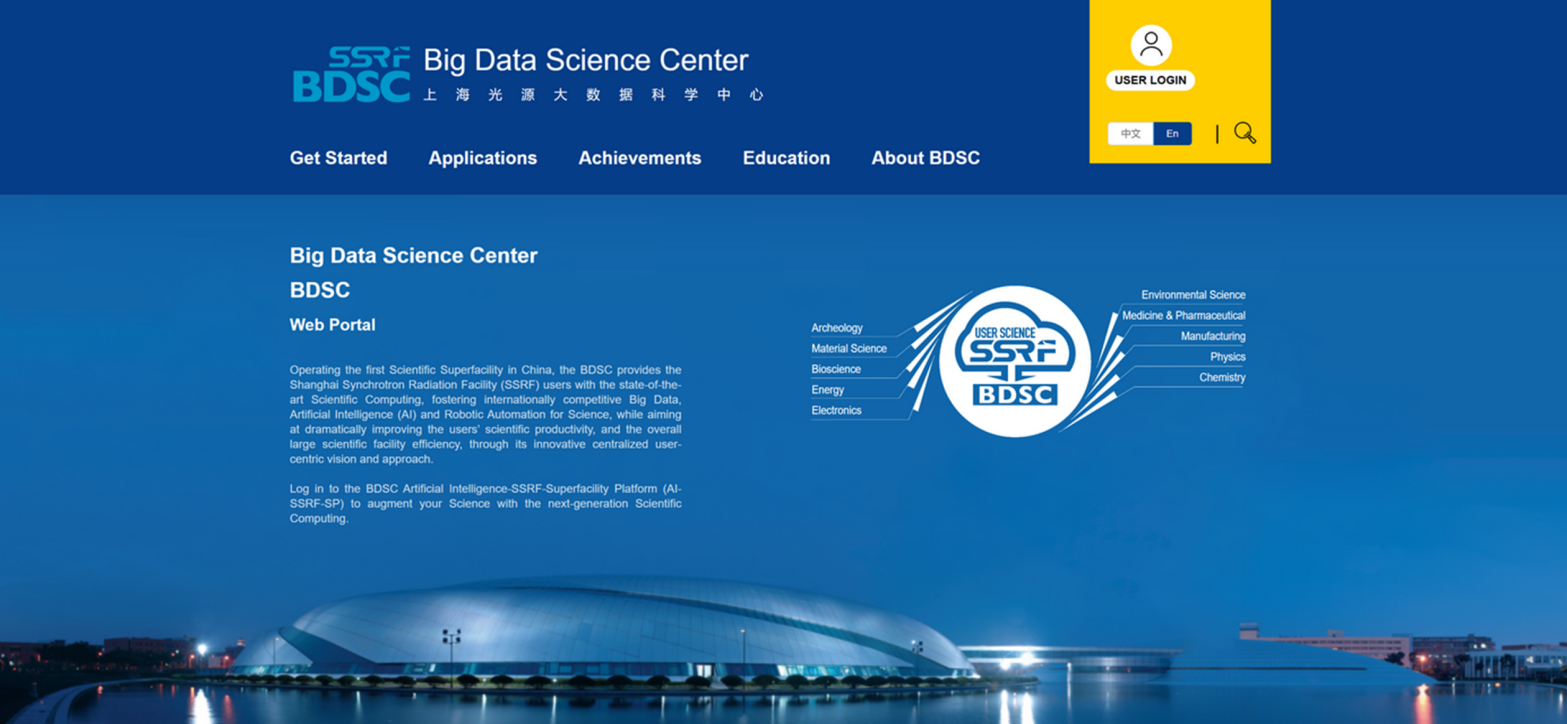
The BDSC web portal.

The AI-SSRF-SP has been developed, deployed and upgraded by the BDSC to address the fundamental challenge of integrating the facility’s heterogeneous data, computational resources and administrative services into a single, intuitive and intelligent portal, thereby transforming a collection of advanced infrastructure into a cohesive, user-centric superfacility experience. In order to enhance the user-centric experience and productivity, the AI-SSRF-SP has been equipped with a multilingual web portal (Fig. 5 ▸).

**Figure 6:**
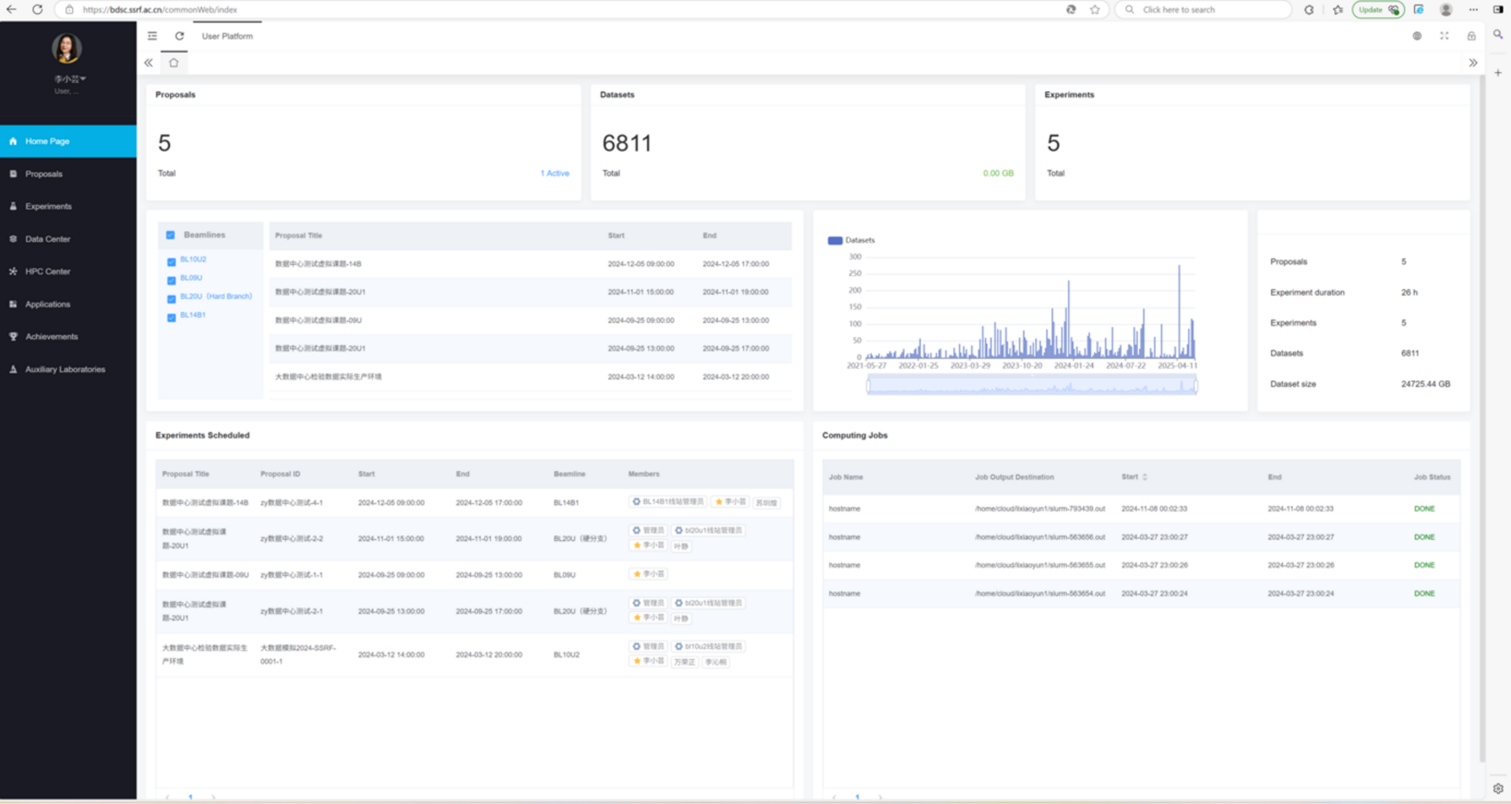
The AI-SSRF-SP user’s personal dashboard functions as a central hub, thoroughly summarizing, through big data technologies, the array of functionalities provided by the AI-SSRF-SP for SSRF users. This comprehensive overview not only highlights the AI-SSRF-SP’s capabilities but also facilitates user navigation and understanding of its features.

This enables users to access their personal page on the AI-SSRF-SP (Fig. 6 ▸), while providing them with comprehensive information regarding the BDSC and the AI-SSRF-SP, including their features, infrastructures, staff, the real-time status of the computing facilities throughout the entire SSRF, a guideline covering the entire SSRF and BDSC workflow, the AI-SSRF-SP scientific cloud with all its applications, the BDSC major achievements, as well as its contributions in terms of national and international training, education, dissemination and collaborations. It is therefore possible for users to access the AI-SSRF-SP using its U^2^IMS in a convenient manner via the web portal. Additionally, a user profile, role and application management framework has been implemented, adhering to the highest security standards, where each account has been further categorized within the AI-SSRF-SP with specific policies in place. Consequently, users belonging to different categories have access to distinct AI-SSRF-SP features, depending on their level of permissions. For instance, the standard user account is only able to access the proposals, experiments and datasets that are associated with the research group to which the user belongs. Furthermore, through the conveniently centralized AI-SSRF-SP, users can directly submit computing tasks to the BDSC HPC infrastructure, access the applications on the AI-SSRF-SP scientific cloud, request access to the SSRF auxiliary laboratories and their equipment, and list their published research results, which are conveniently linked to their experimental data, thus further promoting the implementation of the FAIR principles. Conversely, the beamline administrator account has the capacity to access all proposals, experiments and datasets associated with its beamline, while the administrator account can be granted access to the overall AI-SSRF-SP administration, including the management of users and applications, as well as the monitoring and control systems of the edge computing frameworks.

The AI-SSRF-SP personal dashboard (Fig. 6 ▸) provides a comprehensive overview of the most pertinent information related to the user’s current account. This includes the user’s proposals, their datasets, the arrangements for their experiments, the computing jobs and the HPC resources they have allocated. For each item, the corresponding details can be conveniently visualized with a click.

Furthermore, the AI-SSRF-SP integrates a secure network environment for the data collection that occurs directly at the beamlines, thereby establishing a robust base in terms of user data security. Within the AI-SSRF-SP, a passport for experimental data collection at beamlines is assigned to each proposal. All authorized members of the research group are able to access passport information once they have successfully completed the login process to the AI-SSRF-SP (Fig. S1 of the supporting information). Consequently, in the instances where users employ the corresponding passport provided by the AI-SSRF-SP prior to data collection, their data will be stored in a designated storage space and linked to the relevant proposal. The principal investigator (PI) is enabled to utilize the member management module to add or delete members from the relevant proposals, in addition to granting members the rights to view and download the data associated with the proposal. Moreover, the rate of proposal completion is displayed for the convenience of the users.

Each user’s experiment for each proposal will be displayed and detailed in the AI-SSRF-SP user experiment page, which further fully integrates the SSRF user office system as a module, so that the users can conveniently access all their experiment information from within the AI-SSRF-SP unified and centralized platform (Fig. S2). In this section, users have ready access to a plethora of information relevant to their experiment at the SSRF, including the approval status, the beamline where the experiment is to take place, and the duration and schedule of their granted beam time. In addition, a user-friendly integrated calendar is available, enabling users to conveniently visualize all their scheduled beam times. All the information is stored as metadata and linked to each other, and in turn to the entire users’ research lifecycle, thereby leveraging the full potential of big data, while making them ready for AI training.

The users’ dataset pertaining to the proposals can be viewed in detail, explored or downloaded from the user’s personal data center page (Fig. S3). The AI-SSRF-SP automatically generates a storage folder for the user’s experimental data based on the user’s experiment date and member ID. Each group member is allotted a distinct storage space where their experimental data are saved. The metadata associated with each experiment is then collected, collated and visualized, encompassing both the experiment itself and the user proposal. This comprehensive approach is instrumental in rendering the platform ready for AI applications.

In the case of the beamline’s data analysis pipeline being deployed on the BDSC, the users’ jobs will be automatically submitted directly to the relevant BDSC HPC clusters via their pipeline. SSRF users can then graphically monitor in real time their task and results from their user’s personal HPC center page (Fig. S4), where they can further access a wealth of information, including all the details regarding the HPC resources made available for their job. In addition, a command line interface has been integrated directly into the platform, conveniently accessible from the HPC center page. This feature enables experienced users to interact with the HPC job management system, facilitating operational efficiency and customization. A data browser is also integrated in the HPC center page to allow the user to conveniently explore their data in one place from the inside of the centralized and unified AI-SSRF-SP, and efficiently select those data to be submitted and processed by the BDSC HPC clusters. Similarly, in this case, the metadata are also collected and collated with the remainder of the users’ research lifecycle for the purpose of AI training.

Following approval from the SSRF and BDSC responsible, users are authorized to utilize the software applications via the centralized and unified AI-SSRF-SP scientific cloud, with each software deployed on different HPC nodes. These software applications are comfortably accessible from the user’s personal application page by clicking on the desired application, which are conveniently displayed with graphical interactive and informative icons (Fig. S5). In this instance, a data browser is also integrated into the page with the objective of enabling users to efficiently and conveniently select the data they wish to analyze using the relevant AI-SSRF-SP cloud applications on the relevant BDSC HPC clusters. This entire process occurs within the same centralized and unified AI-SSRF-SP. In addition, several native and virtualization approaches are integrated within the AI-SSRF-SP cloud and leveraged to provide virtualization environments to those software applications that require it. This enables centralized deployment and concurrent real-time management of complex software, remote graphical streaming for highly interactive sessions, and secure access control to ensure system and data integrity. In this case, the metadata are also collected and collated with the remainder of the users’ research lifecycle for the purpose of training an AI on the critical aspect of scientific software applications usage, best practices and optimization.

Users are also able to link their achievements, publications and patents with the relevant beamlines and proposals via their personal achievement page (Fig. S6). All the dataset are linked to the corresponding proposals, thus enabling the AI-SSRF-SP to provide traceability for the experimental data and link them to the corresponding publication. Similarly, the metadata are also collected and collated with the remainder of the users’ research lifecycle for the purpose of training AI to discriminate the quality of research based on its scientific outcomes. This is of crucial importance to implement AI-guided best practices in setting up experiments at large-scale facilities, tuning and optimizing scientific software and computing resources tailored to the specific needs of each experiment.

The AI-SSRF-SP has also been integrated and provides users with full access to the SSRF Experimental Auxiliary System through the user’s personal auxiliary laboratory page, all within the same centralized and unified AI-SSRF-SP (Fig. S8). This encompasses the integration of the auxiliary laboratory for material sample preparation, *in situ* studies, chemistry and environmental science, and biology and medical sciences within the AI-SSRF-SP. Consequently, users are able to access a comprehensive overview of the auxiliary laboratory resources made available to them by the AI-SSRF-SP, and which are associated with their respective proposals. Furthermore, it is possible for users to make bookings for any instruments available at the SSRF auxiliary laboratory via this dedicated AI-SSRF-SP section. In addition, the status of requests can be checked and reservations can be consulted visually through a convenient calendar that is integrated directly into this section. It is also possible for users to verify their equipment usage quota and the corresponding payment bills. Furthermore, this section enables the user to peruse all the announcements from the auxiliary laboratory responsible, as well as to access all the manuals, instructions and guiding materials in a centralized manner. Consequently, the auxiliary laboratory reservation system is fully integrated directly into the AI-SSRF-SP (Fig. S9), thereby providing the user with a comprehensive overview of each instrument.

The successful implementation of the AI-SSRF-SP platform establishes a high-level unifying wrapper for the entire scientific workflow, demonstrating its effectiveness as a central hub that streamlines research activities, enforces FAIR data practices, and organizes metadata in an AI-ready manner across the user’s proposal-to-publication lifecycle. To further enhance user research efficacy and promote the deep integration of large language models (LLMs) with the specialized field of synchrotron radiation, the BDSC has initiated the development and deployment of an intelligent experimental assistance system at the SSRF, enhanced by LLMs and knowledge graphs, leveraging the DeepSeek and Qwen frameworks. Focused on core functionalities such as experimental plan co-design, accelerator/beamline domain-specific Q&A agents, and online experimental operation guidance, this system aims to address complex scenarios within synchrotron-radiation-focused AI for science. Moreover, it is designed to provide a foundational platform to support further research on AI agents.

### 3.4. Remote experiments

To addresses the challenge of circumventing geographical and logistical constraints by enabling users to perform complex synchrotron experiments entirely off-site, necessitating a secure, reliable and efficient remote operation framework that extends beyond simple data transfer to encompass real-time experimental control, the BDSC has implemented a remote experiment framework integral to the superfacility infrastructure at the SSRF.

**Figure 7:**
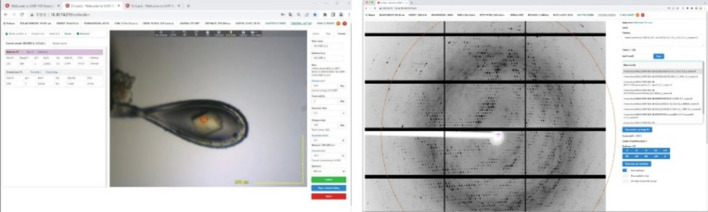
A user’s remote experiment running at the SSRF leveraging the web application Finback.

The remote experiments are mainly carried out at the SSRF beamlines supporting the users’ investigations on proteins (*i.e.* BL02U1, BL10U2, BL18U1 and BL19U1). The users remotely access the web-based data processing platform through the BDSC VPN. They deliver their samples to the SSRF, at least one day prior to their scheduled remote experiment. Locally assisted by the SSRF engineers at the beamlines, the users can remotely collect, view and automatically process their data, leveraging the Finback web application (Yu *et al.*, 2024 ▸) (Fig. 7 ▸). The BDSC provides the SSRF users with the highest security standards, therefore the remote experiments are carried out using only the strictly necessary applications, and without any remote desktop. After that the remote experiments are carried out; the SSRF users can use the web application to download their data.

The implementation of a secure, application-specific remote access system for key beamlines represents a pivotal advancement in user service, successfully transforming traditional, location-bound experiments into remotely operable workflows and significantly expanding the large facility’s reach and operational flexibility.

## 4. Conclusion

SSRF has launched a comprehensive digitalization and intelligent upgrade project aimed at leveraging AI for the operation of its accelerators, beamlines and experimental stations, in addition to the analysis and processing of scientific data. Within this initiative, the BDSC will focus on several key areas: the structured integration and annotation of multi-source scientific data, the multimodal fusion analysis of machine data and scientific data and the development of an AI data foundation and a model foundation. These efforts are designed to support and enable intelligent and autonomous experimental control and facilitate autonomous scientific discovery.

As outlined in the article, the achievements made have contributed to the integration of scientific computing, big data large-scale frameworks, HPC and data science in synchrotron facilities within a unified and centralized platform. This integration has the potential to significantly influence future research directions in multidisciplinary science, including, but not limited to, chemistry, physics, energy and material science, medical and pharmaceutical science, bioscience, electronics, environmental sciences, archeology and manufacturing. The article emphasizes the role of the AI-SSRF-SP, as the high-level unifying and centralizing wrapper for scientific computing, big data large-scale frameworks and HPC, in enabling complex simulations and data analysis, which are essential for understanding intricate systems. As computational tools become increasingly sophisticated and more seamlessly integrated with AI, they will empower researchers operating within large-scale scientific facilities, equipped with intelligent, unified and centralized scientific computing platforms, to conduct more predictive simulations, thereby minimizing the necessity for extensive experimental trials. This shift has the potential to enhance the efficiency of research procedures, whereby theoretical predictions inform experimental designs, thereby accelerating the pace of discovery in the most diverse research fields. By leveraging large datasets generated from synchrotron experiments, researchers can develop predictive models that enhance the understanding of chemical and biochemical reactions and material properties. This integration not only streamlines data analysis but also opens new avenues for discovering novel compounds and optimizing synthetic pathways, which are crucial for drug discovery and environmental applications. Furthermore, our findings advocate for increased collaboration between scientists, data scientists and computational researchers. As the complexity of physical, chemical and biological systems grows, interdisciplinary approaches will be necessary to tackle these challenges effectively. The article suggests that fostering such collaborations can lead to innovative solutions and methodologies that enhance research outcomes across various scientific subfields. Therefore, the emphasis on AI-enabled big data analytics and the adoption of FAIR data practices will likely shape future research frameworks. By ensuring that data generated from synchrotron facilities are well managed and accessible through a unified and centralized platform, researchers can build upon each other’s work more effectively, leading to cumulative advancements in the field. This approach can also facilitate the development of standardized protocols for data sharing and analysis, which is essential for collaborative research efforts. Furthermore, the scalability of the scientific computing solution presented in the article is both timely and relevant in light of future research directions, which stand to benefit significantly from the advancement of integrated quantum-HPC exascale platforms. These platforms have the potential to further enhance the computational capabilities tailored for scientific research at large-scale scientific facilities. This integration is expected to unlock new possibilities for solving complex multidisciplinary problems, particularly in areas like quantum-scale science, materials design and drug discovery. As these technologies evolve, they will likely redefine the landscape of scientific research, making it imperative for scientists to seamlessly adapt and embrace these advancements through facility-wide unified and centralized platforms. Indeed, the AI-SSRF-SP draws attention to the critical role of centralizing scientific computing, big data frameworks, tailored HPC architectures, data science and experimental data within a unified platform, in shaping the future of multidisciplinary and multimodal research. By enhancing and facilitating user-friendly and user-centric access and usage of scientific computational capabilities, integrating AI, fostering interdisciplinary collaboration and promoting data management practices, the international scientific community can expect to see significant advancements in both fundamental and applied research.

## Supplementary Material

Sections S1 to S4, including Figs. S1 to S9 and Table S1. DOI: 10.1107/S1600577526005795/ye5077sup1.pdf

## Figures and Tables

**Table 1:** A summary of the BDSC software environments at the SSRF

Operating system	Linux Red Hat 64-bit
Compiler	GNU C / C++ / F77 / F90 Intel C / C++ / Fortran
Mathematical library	Intel MKL / FFTW2 / FFTW3 / Openblas / Blas / LAPACK / ScaLALACK
Parallel environment	OpenMPI / Mvapich / Mpich
GPU environment	CUDA / cuDNN
Cluster management and scheduling system	CHESS EaaS Platform
Other software environments	Perl / Python / Java / PyTorch / TensorFlow / Anaconda / Xcode
Scientific software	LAMMPS / GROMACS / MATLAB / Gaussian / VASP / Ansys / SOLIDWORKS / SIMON / Igor / Avizo / CASTEP / MuMax / Quantum ESPRESSO / SIESTA / CP2K / BigDFT / Siam Quantum / VMD / Crystal / PWscf / CT Reconstruction Packages / Jmol / RasMol / PyMol / AtomEye / OVITO / GFN2-xTB

**Table 2:** Remote file transfer efficiency of the BDSC VPN at the SSRF

Remote transfer	Efficiency	
File type	Upload rate	Download rate
Tiny file (2.3 MB)	21 MB/s	10–20 MB/s
Small file (10 MB)	5 MB/s	10–20 MB/s
Large file (2.6 GB)	6.5 MB/s	20 MB/s

## Data Availability

The BDSC web portal is accessible at https://bdsc.ssrf.ac.cn/en. The code for the AI-SSRF-SP is available at https://doi.org/10.6084/m9.figshare.29275790.
