# Supplementary material for: The Big Data Science Center at the Shanghai Synchrotron Radiation Facility: the architecture of the superfacility
Source: J Synchrotron Radiat. 2026 Jun 22;33(Pt 4):939–52. doi: 10.1107/S1600577526005795 (PMC13344637; doi:10.1107/S1600577526005795)
Supplement: Supplementary file 1 [file s-33-00939-sup1.pdf]

JOURNAL OF SYNCHROTRON RADIATION

Volume 33 (2026)

Supporting information for article:

The Big Data Science Center at the Shanghai Synchrotron Radiation Facility: The Architecture of the Superfacility

Xiaoyun Li, Chunpeng Wang, Jige Chen, Rongzheng Wan, Jing Ye, Qintong Li, Haojun Cai, Ying Zhang, Aiguo Li, Renzhong Tai and Alessandro Sepe

S1. High throughput data pipelines and massive storage system

The BDSC has architected a storage infrastructural deployment plan divided into two phases: Phase I, deploying the Huawei OceanStor 9000; and Phase II, deploying the OceanStor 9550, and, thus, upgrading the Phase I capabilities. A NAS cluster, comprised of 39 nodes, constitutes the BDSC file storage. Each node comprises Serial Advanced Technology Attachment (SATA) and Solid State Disk (SSD) disks, providing 22.5 PB of combined total storage. The WushanFS distributed file system is the core component of the OceanStor series. It integrates the disks from all nodes into a unified resource pool, while providing a unified namespace. At the same time, it provides with cross-node, cross-rack and different levels of data redundancy, which protects the users' data, guaranteeing high availability while handling high disk utilization, thus, avoiding the traditional storage stack disadvantages. The storage system provides Common Internet File System (CIFS) and Network File System (NFS) access functionalities, equipped with a unified domain name access mechanism, thus, enabling the SSRF users to seamlessly access the unified storage system.

The BDSC storage system uses the Windows Active Directory (AD) for the identity authentication, and allocates storage space for each user. Users can mount the storage that they require on either Windows or Linux clients and access their own data regardless of which terminal they use at the SSRF.

S2. Robotic data archiving and backup

The Dell PowerVault ML6000 series tape system is used to back-up all the data from the BDSC systems and the users' experiments. Equipped with scalable on-demand capacity expansion functionalities, the available capacity can be easily increased without interruption. An enterprise-class tape system is capable of providing multiple tape drives and hundreds of tape slots. A robotic arm automatically disassembles and loads tapes. While providing basic automatic data backup and recovery functions, it also brings more technological advanced features. It can work in parallel with multiple drives, or it can have several drives pointing to different servers for backup, it can store PB of data, while providing continuous backup, automatic tape search and other functions, and it can be supported by an intelligent management and recovery software, further providing real-time monitoring and statistics; thus, resulting in being the main device for the centralized network data backup.

S3. Online real-time user data analysis

The BDSC HPC infrastructure is comprised of the HPC I and HPC II systems. The HPC I is based on a Huawei E9000 blade server system, a half-width CH121 blade housing the CPU compute nodes, and a full-width CH220 housing the GPU compute nodes. The HPC II uses a Huawei micro-module

system and the Inspur i24M6 2U4N high-density blade for the CPU compute nodes, the H3C R5300 blade for the GPU compute nodes and the H3C R8900 blade for the FAT nodes. The E9000 offers top-class high-density computing and memory architecture, as well as a comprehensive modular design for power supply, cooling, management, switching, etc. In terms of reliability, the E9000 adopts full component-level redundancy to ensure that, in the unfortunate case of a single point of failure, the services remain available. Additionally, the compute nodes are provided with fault tolerance, ensuring protection in the unfortunate case a single CPU or memory module would fail. The HPC I provides the SSRF users with the access to 1 FAT node and 48 CPU nodes, which include 4 dual-CPU nodes connected with PCIe and 5 GPU nodes with 12 NVIDIA Tesla P100 graphics cards, including 1 quad-GPU node connected with NVLINK. The BDSC, subsequently, upgraded its HPC framework, adding a second HPC system, the HPC II, which provides the SSRF users with the access to 160 CPU nodes, 2 FAT nodes and 8 GPU nodes, which include 4 dual-CPU nodes connected with PCIe (both for CPU and FAT nodes), 4 GPU nodes with 16 NVIDIA A100 graphics cards and 4 GPU nodes with 32 Hygon K100_AI graphics cards. The theoretical peak for CPU computing power of the HPC I is 143.18 TFlop/s, with the measured operating efficiency of the Linpack test being ≥ 70% (as shown in Table S1); the theoretical peak of the GPU computing power of the HPC I is 58.8 TFlop/s, with the measured operating efficiency of the Linpack test being ≥ 68.93% (as shown in Table S1). Meanwhile, the theoretical peak of CPU computing power of the HPC II is 615.5 TFlop/s, with an operational efficiency ≥ 52%; the theoretical peak of the GPU (NV cards) computing power of the HPC II is 155.2 TFlop/s, with an operational efficiency ≥ 55%, and the theoretical peak of the GPU (Hygon Cards) computing power of the HPC II is 1560 TFlop/s, with an operational efficiency ≥ 76%.

Table S1 HPC cluster benchmarks at the BDSC.

Cluster		Nodes	Specification	Theoretical peak (Tflops)	Linpack test (Tflops)
HPC I	CPU	cpu01-48	Gold 6140	127.18	87.10
		agpu01-agpu04	Gold 5118	3.53	2.30
		bgpu01	Gold 6132	2.33	1.60
		Fat01	E7-8860	10.14	9.23
		Total		143.18	100.30
		Linpack test measured operating efficiency ≥ 70%			
	GPU	agpu01-agpu04	Tesla P100 PCIe	37.60	25.53
		bgpu01	Tesla P100 SMX2	21.20	15.00
		Total		58.80	40.53
		Linpack test measured operating efficiency ≥ 68.93%			
HPC II		CPU CPU1-160	Gold 5320	586	304.7
		FAT01-02	8260	29.5	14.8

	Total		615.5	319.5
	GPU GPU01-04	A100	155.2	85.3
	GPU GPU05-K08	K100_AI	1560	1200
		Linpack test measured operating efficiency ≥ 52% (NV) and ≥ 76% (Hygon)		

S4. Facility-wide asymmetric and high-concurrency computing architecture

The 20 edge clusters deployed by the BDSC at the SSRF beamlines account for 18 CPU nodes (4 nodes with AMD EPYC 7702 x 2 per node, 12 nodes with Intel Xeon 6226R x 2 per node and 2 nodes with Zhaoxin ZX-E KH-3800D x 2 per node) and 2 GPU nodes (NVIDIA RTX 5000 x 2 and RTX 3080Ti).

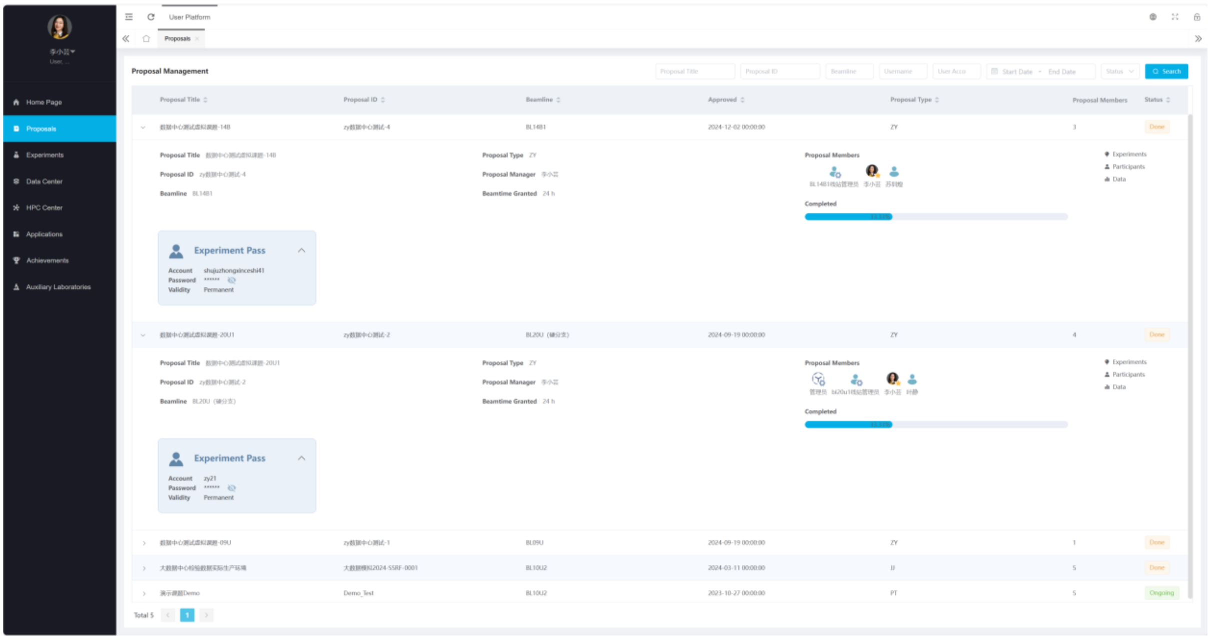

Figure S1 The AI-SSRF-SP user's personal proposal page. The proposal page is intended to provide a comprehensive overview of all user proposals, incorporating features such as the experiment passport and functions to authorise the participation of new group members in proposed research projects.

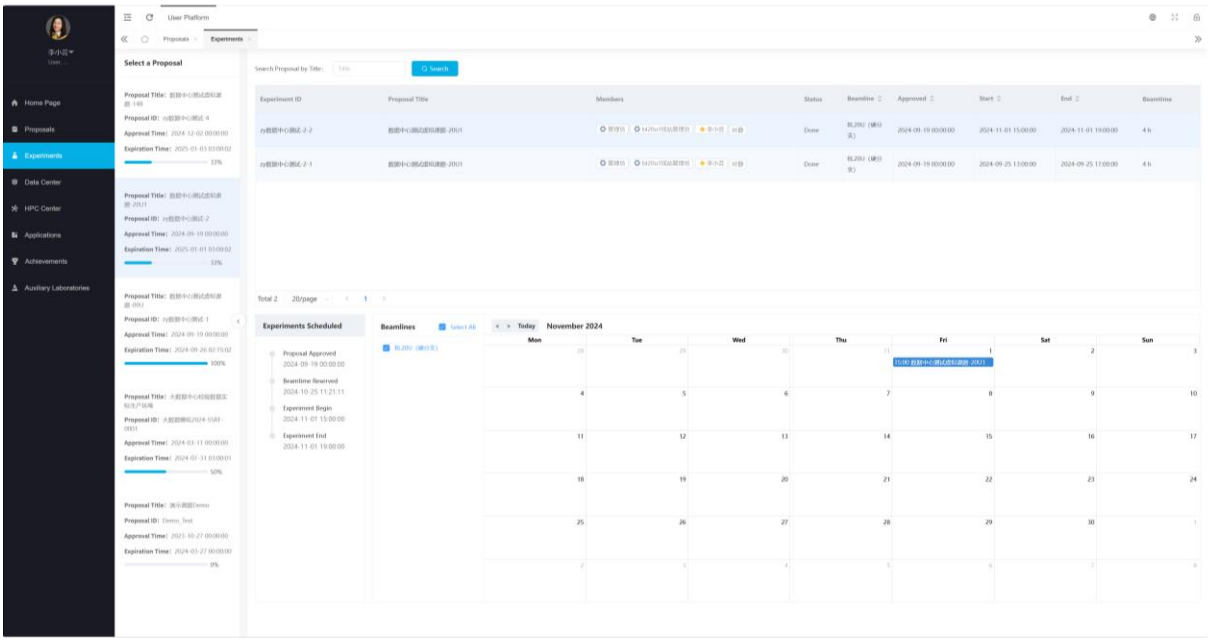

Figure S2 The AI-SSRF-SP user's personal experiment page.

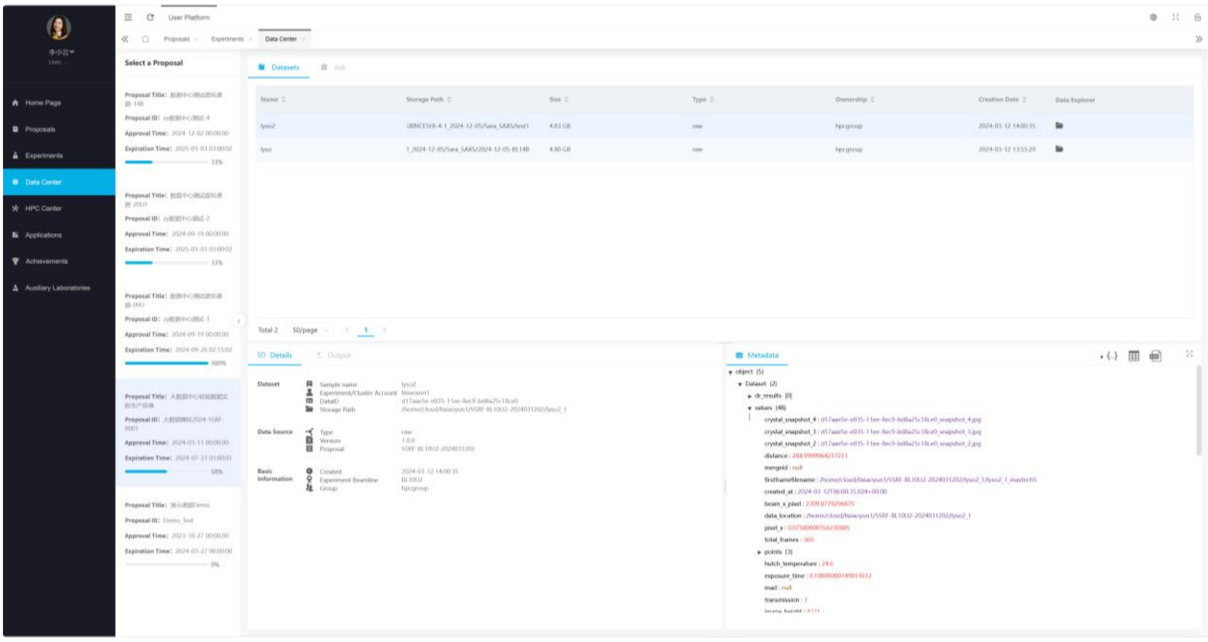

Figure S3 The AI-SSRF-SP user's personal data center page.

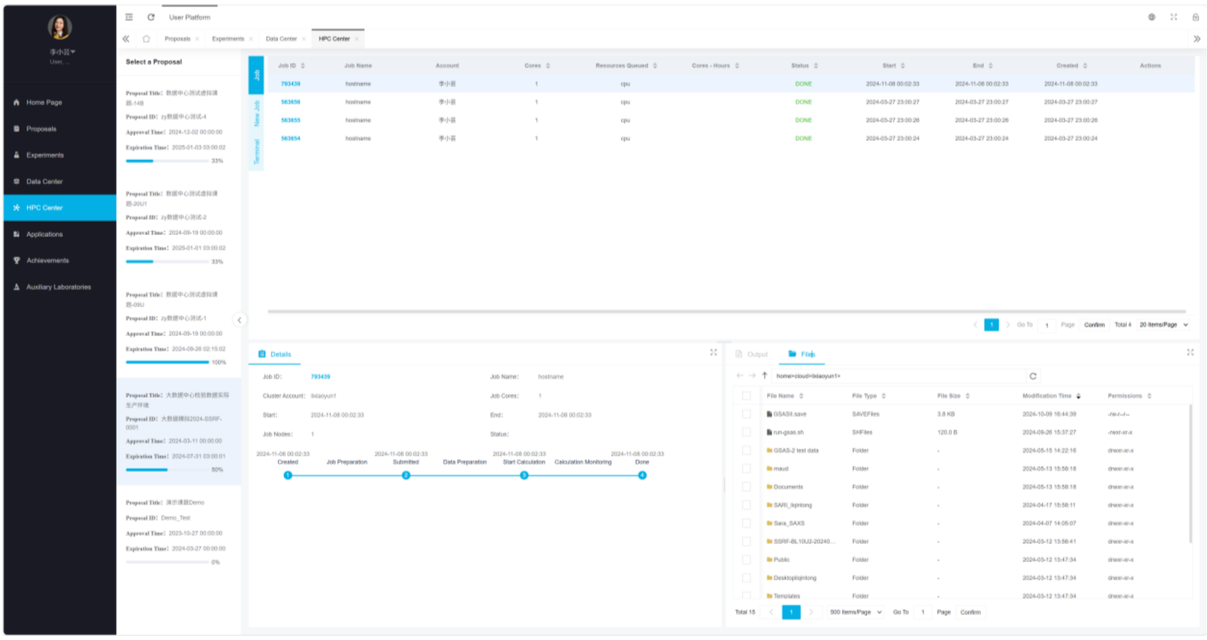

Figure S4 The AI-SSRF-SP user's personal HPC center page.

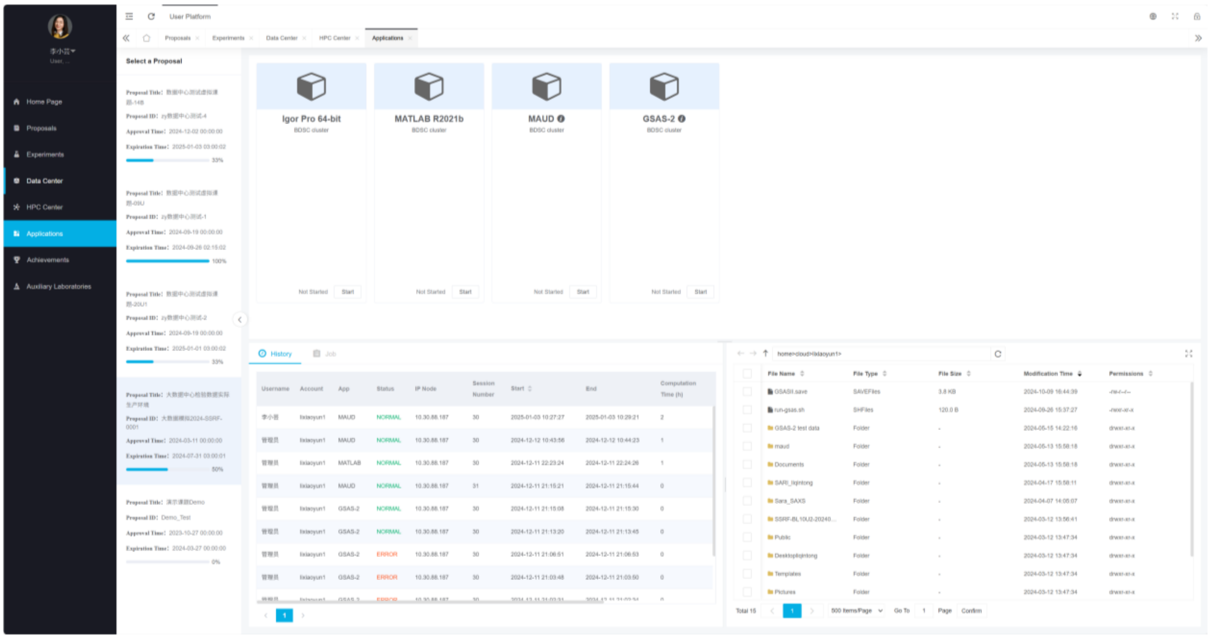

Figure S5 The AI-SSRF-SP user's personal application page.

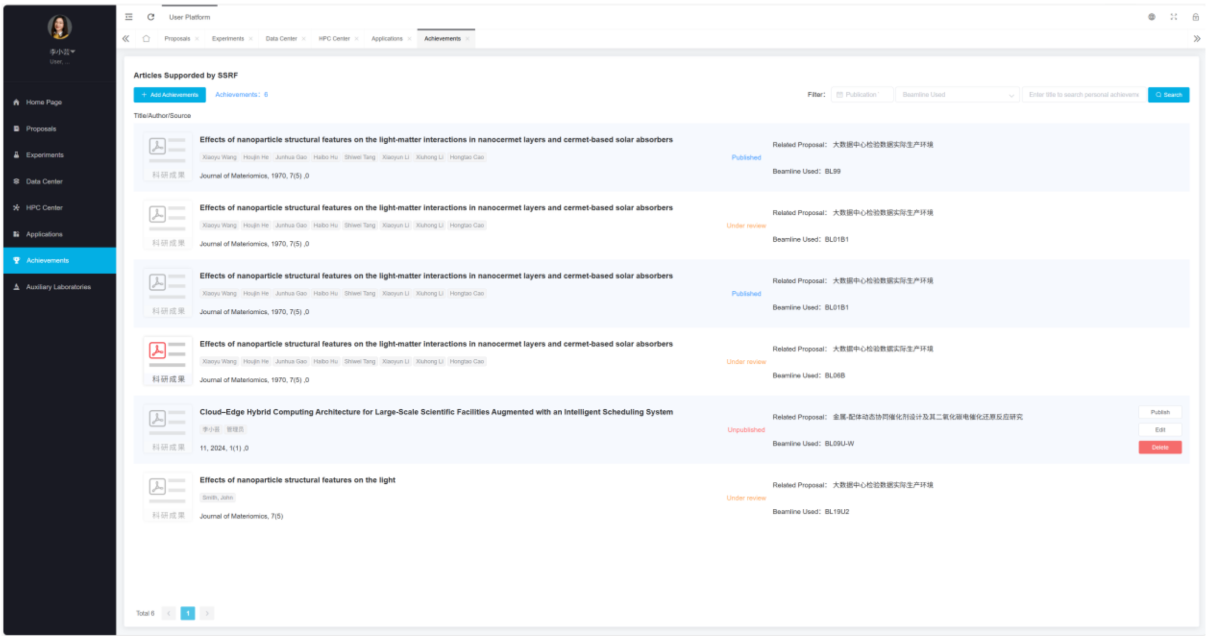

Figure S6 The AI-SSRF-SP user's personal achievement page, encompassing all the modules and functions necessary to integrate their research achievements from both internal and external sources.

Users are prompted to provide all the relevant information regarding their achievements through different methods, which are encoded into different APIs within the AI-SSRF-SP, so that they are imported into the AI-SSRF-SP: i) through the searching module, which allows a scientific publication to be discovered through its DOI, ii) through the information stored within the SSRF user management system, iii) through the paper's citation, or iv) manually inputting all the relevant information (Fig. S7).

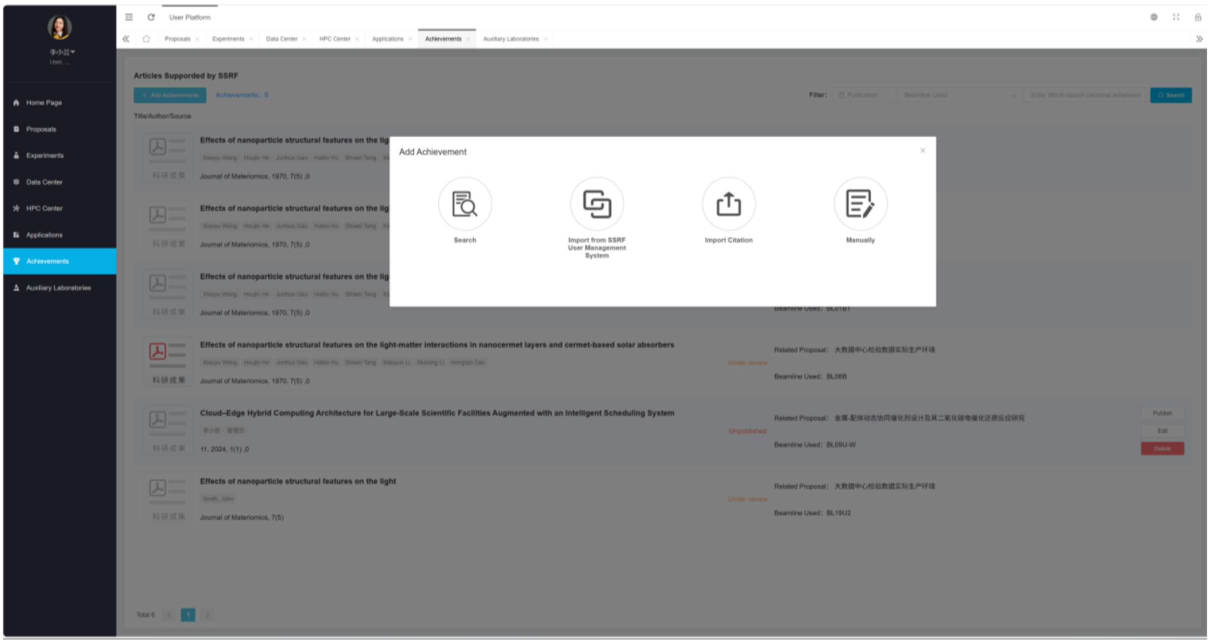

Figure S7 Functional module, integrated into the AI-SSRF-SP users' personal achievements page, and designed to facilitate users in the submission of their research achievements.

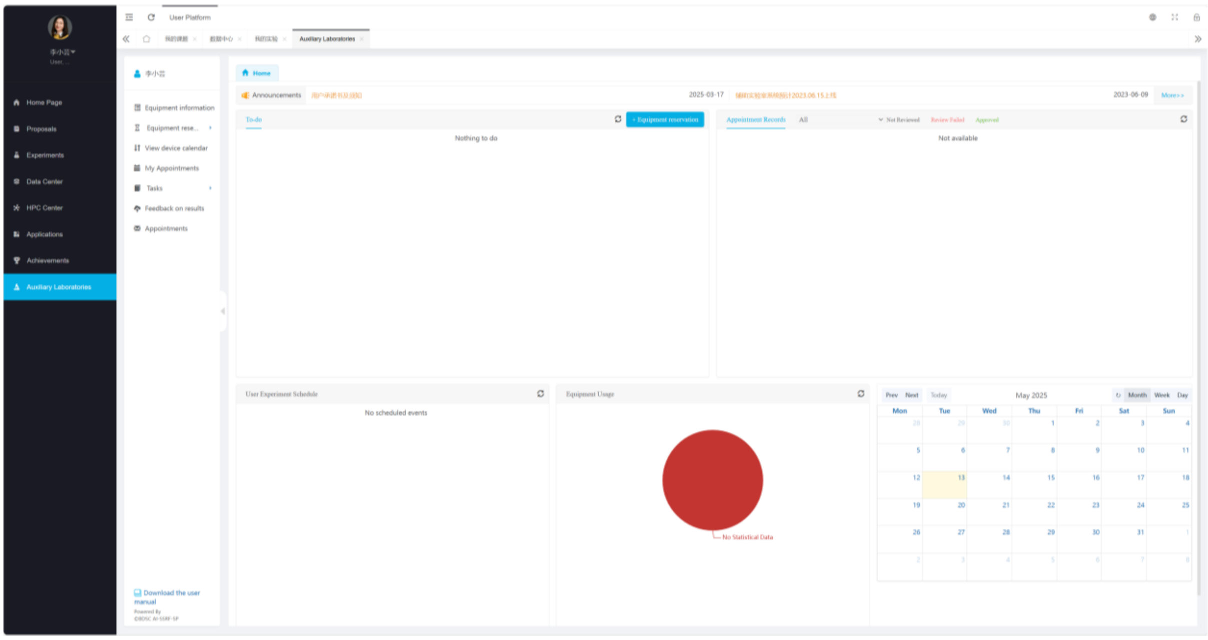

Figure S8 The AI-SSRF-SP user's personal auxiliary laboratory page.

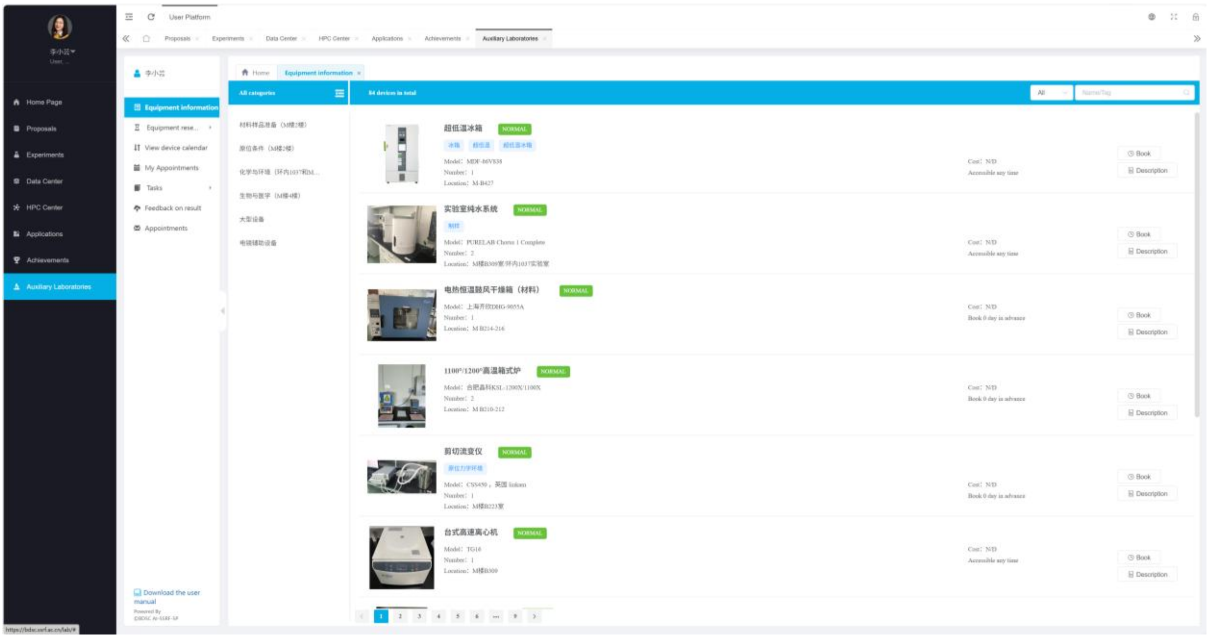

Figure S9 The auxiliary device booking system, including device descriptions, is integrated into the AI-SSRF-SP user's personal auxiliary laboratory page. This encompasses a general description, technical specifications that are intended to facilitate the selection of the most suitable equipment for the proposed experiment by the user, the number of available instruments, and their respective locations. Furthermore, within the AI-SSRF-SP, the equipment is systematically categorised in a manner that facilitates efficient review of its suitability for the user's experiment and rapid identification of the equipment based on optimal fit with the experimental needs of the user.
